# Leptomeningeal Metastatic Prostate Cancer Imitating a Subdural Hematoma

**DOI:** 10.7759/cureus.31380

**Published:** 2022-11-11

**Authors:** Behrouz Zamanifekri, Mani Zamanifekri, Shahab Babakoohi, Mark Whalen, Alireza Minagar

**Affiliations:** 1 Neurology, Caromont Health, Gastonia, USA; 2 Biology, University of South Carolina, Columbia, USA; 3 Oncologic Dermatology, Atrium Health Wake Forest Baptist, Winston-Salem, USA; 4 Pathology, Caromont Health, Gastonia, USA; 5 Biotechnology (Bioinformatics), University of Maryland Global Campus, Adelphi, USA

**Keywords:** mri in dural metastasis, leptomeningeal metastases, advanced prostate adenocarcinoma, dural metastasis, subdural hematoma (sdh), metastases to dura

## Abstract

Metastases to the dura and adjacent parenchyma occur in 1%-2% of patients with prostate cancer. Dural metastases manifest as subdural fluid collections and present as a chronic subdural hematoma. We present a patient with advanced prostate cancer who experienced a fall and a traumatic brain injury. Computer tomography (CT) of the brain revealed a bilateral subdural hematoma (SDH). During the follow-up examination, the patient’s mental status declined, and a follow-up brain CT showed an expansion of the SDH. Brain MRI with contrast demonstrated dural lesions suspicious for metastasis to the dura. Histopathologic examination of the lesions confirmed metastatic prostate adenocarcinoma.

While uncommon, leptomeningeal-dural metastatic lesions stemming from prostate adenocarcinoma should be suspected in patients with known prostate cancer who present with subdural collections. This is even more significant if the patient with prostatic adenocarcinoma has sustained a recent head injury and presents with a subdural hematoma on neuroimaging. Brain MRI provides more data towards the differential diagnosis of these lesions and should be an essential part of the diagnostic workup. Biopsy and histopathologic examination of these lesions are indicated in the diagnostic workup of these uncommon lesions.

## Introduction

Prostate adenocarcinoma is the most common cancer and the third most common cause of death due to cancer among males aged 80 years and older [1]. Globally, there are an estimated 1,400,000 new cases of prostate cancer annually, making it the second most commonly diagnosed cancer in men [2].

The clinical course of prostate cancer is quite variable, ranging from low-grade and indolent lesions to high-grade, aggressive, and metastatic lesions at the time of diagnosis.

The staging system is based on the extent of the primary tumor (T), involvement of the lymph node(s) (N), and the presence or absence of metastasis (M), along with the measurement of serum levels of prostate-specific antigen (PSA) and the histologic grade of the primary tumor [3].

Prostate adenocarcinoma constitutes the most common type of prostate cancer and frequently metastasizes to the regional pelvic structures (i.e., rectum, bladder, seminal vesicles, pelvic wall) and lymph nodes (sacral, obturator, iliac) and then to the bone, liver, and lungs. The bones of the axial skeleton are the predominant site of metastasis [4]. Metastasis to the central nervous system (CNS) is rare, with an incidence of up to 4% to the brain and dural involvement in 1%-2% of cases. Interestingly, dural metastasis originating from prostate adenocarcinoma is more common in cases that are refractory to hormone-based therapy [5].

## Case presentation

**Figure 1:**
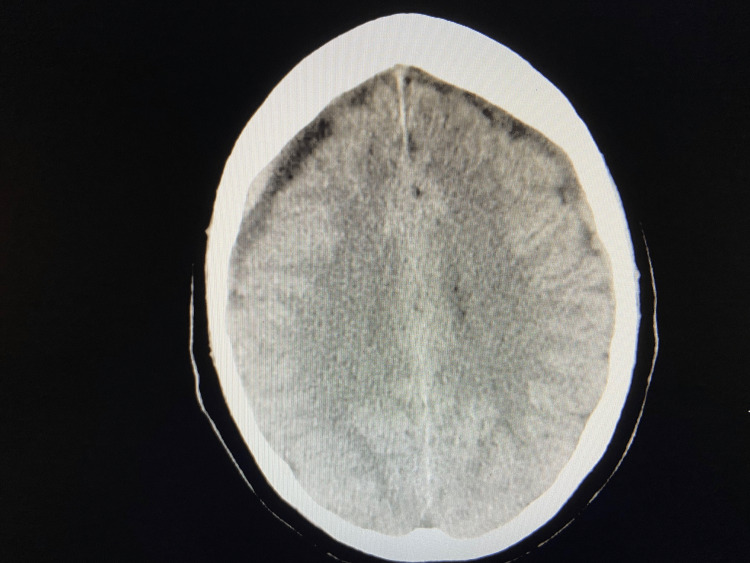
A CT of the brain (without contrast) showed bilateral subdural hematomas within the lateral convexities

A 65-year-old African American male presented to the emergency room with a worsening headache and fatigue. Past medical history was significant for diabetes mellitus and castration-resistant prostate cancer with metastasis to the cervical, thoracic, and lumbar spines. Prostate cancer was initially diagnosed at age 56 with a Gleason score of 9.0. The patient had completed radiation therapy and was receiving hormone-based chemotherapy. Three weeks earlier, the patient fell on the ground and sustained head trauma without loss of consciousness. Computer tomography (CT) of the brain without contrast revealed a bilateral subdural hematoma (SDH), and the patient was discharged with no further therapeutic interventions. (Figure 1)

**Figure 2:**
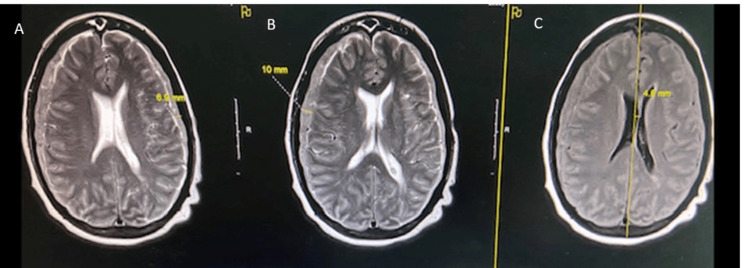
Figures A and B: Brain MRI of the T2 blade (axial view) showing increased signal within the bilateral temporal lobes as well as cortical thickening and edema. Figure C: T2 FLAIR (axial view), showing increased mass effect with a leftward midline shift of 5 mm.

During the next three weeks, the patient’s neurologic status deteriorated with worsening headaches, confusion, and overwhelming fatigue. An MRI of the brain revealed expansion of the bilateral SDH, causing a midline shift, and the presence of hyperintense lesions in the subarachnoid space on T2-weighted and fluid-attenuated inversion recovery (FLAIR) sequences. Brain MRI sequences are presented in Figure 2.

**Figure 3:**
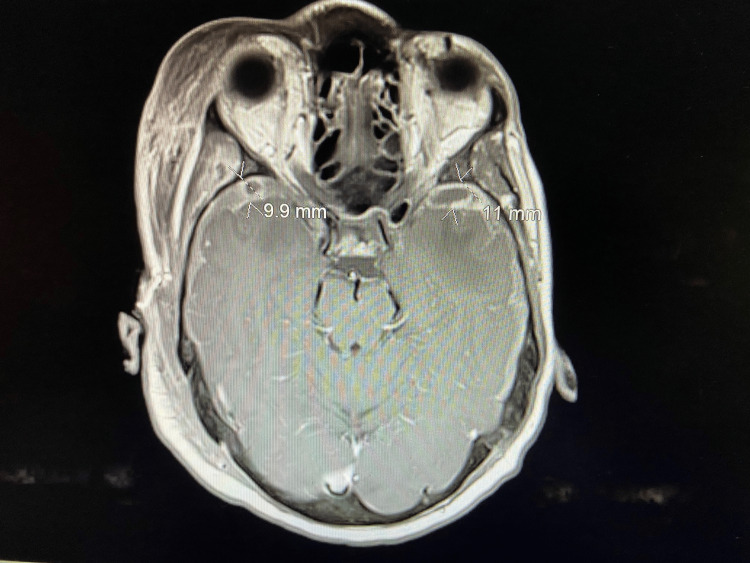
Following a right frontal craniotomy and partial evacuation of a right-sided subdural collection, an MRI of the brain (axial view) with gadolinium contrast revealed areas of subarachnoid hemorrhage in the underlying sulci, restricted diffusion, irregular dural enhancement, areas of vasogenic edema involving bilateral temporal lobes, and diffuse abnormal bone marrow signal intensity.

During the last presentation, laboratory workup revealed a serum PSA level of 3700 ng/mL (range of 0-4 ng/mL) and an alkaline phosphatase level of 450 U/L (range of 42-121 U/L). A craniotomy was performed, and right-sided subdural hemorrhage and collections were partially evacuated. (Figure 3). Findings were mixed blood, pink tissue, and irregular dura. A pink-colored tissue mass measuring 3.0 x 3.0 x 0.6 cm was resected from the subdural space. Neuropathologic examination established a diagnosis of metastatic prostate adenocarcinoma with dural involvement.

**Figure 4:**
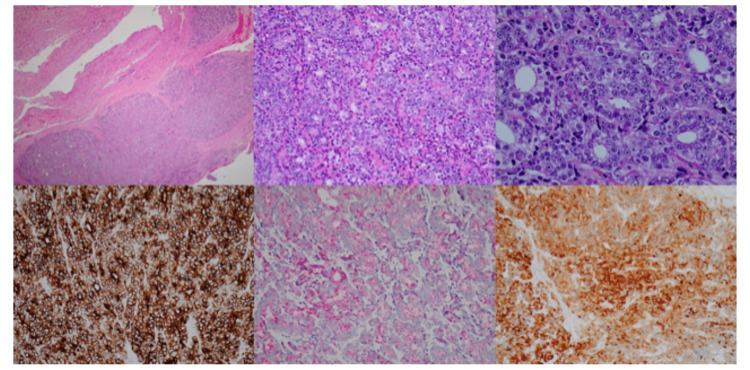
Top-left: H&E stain, 40X, low magnification of the tumor extending into adjacent dural tissue; top-middle: tumor forming sheets of back-to-back malignant glands (adenocarcinoma). H&E stain, 200X; top-right: Close-up of malignant glands, H&E stain, 400X; bottom-left: Positive CAM5.2 immunostain (cytoplasmic positivity); bottom-middle: Positive 504S (also known as AMACR) stain (granular cytoplasmic positivity); bottom-right: Positive PSA immunostain (cytoplasmic staining)

Further examination of the tissue demonstrated positivity for CAM, 5.2, OSCAR, PSA, AMACR, and NKX1A and negativity for cytokeratin, AE1/AE3, CK7, CK20, GFAP, TTF-1, napsin, and CDX2. Histopathologic slides are presented in Figure 4.

## Discussion

Dural metastasis of the prostate adenocarcinoma is rare and may mimic acute or chronic SDH. Direct hematogenous spread, extension from bony skull metastases to the leptomeninges, and, in rare cases, dissemination from cerebral metastases are the mechanisms of metastatic spread. Clinically, patients present with non-specific symptoms such as headache and/or altered mental status [6]. In addition, leptomeningeal metastases, or carcinomatous meningitis, are a rare complication of advanced cancer from solid tumors, most commonly breast cancer (16%), lung cancer (10%-26%), and melanoma (5%-25%). Leptomeningeal metastases are diagnosed in 5% of patients with metastatic cancer [5].

Hemorrhage within the subdural space may stem from a rupture of the vasculature within the metastatic lesions, mechanical obstruction of the dural veins, or hemorrhage from metastatic lesions. Most commonly, a subdural hematoma is diagnosed based on a head CT without contrast. In our patient, with a history of falls and head trauma, the presence of dural metastases was suspected on brain MRI and later confirmed following surgical resection and histopathologic examination of the resected tissue. Brain MRI with gadolinium infusion is a much more sensitive diagnostic tool for the diagnosis of dural lesions and provides significant data regarding the lesion's morphology, its dimensions and extent of growth, and its resectability [6]. The advent of new techniques such as magnetic resonance spectroscopy (MRS) may improve diagnostic capabilities. Ultimately, diagnosis is established and confirmed by histological examination of the resected tissue. The median survival from the diagnosis of dural metastases was three to four months [6].

## Conclusions

Dural metastasis of prostate adenocarcinoma is rare and may mimic SDH. Brain CT is generally not helpful in the diagnosis of dural metastatic lesions, and brain MRI is far superior in showing dural metastasis in patients with advanced prostate adenocarcinoma, even with a history of falls and head trauma. Biopsy and histopathologic examination of the biopsied tissue play significant roles in the diagnostic workup. In addition, radiologically, expansion of the dural lesion on subsequent scans strongly indicates the presence of a metastatic lesion. Patients with advanced prostate adenocarcinoma and dural metastases generally have a poor prognosis.
